# Elevated high-sensitive troponin T on admission is an indicator of poor long-term outcome in patients with subarachnoid haemorrhage: a prospective observational study

**DOI:** 10.1186/s13054-015-1181-5

**Published:** 2016-01-19

**Authors:** Jonatan Oras, Christina Grivans, Andreas Bartley, Bertil Rydenhag, Sven-Erik Ricksten, Helene Seeman-Lodding

**Affiliations:** 1 Department of Anaesthesiology and Intensive Care Medicine, Institute of Clinical Sciences, Sahlgrenska Academy, University of Gothenburg, Gothenburg, Sweden; 2 Department of Clinical Neuroscience, Institute of Neuroscience and Physiology, Sahlgrenska Academy, University of Gothenburg, Gothenburg, Sweden

## Abstract

**Background:**

Patients with subarachnoid haemorrhage (SAH) frequently develop cardiac complications in the acute phase after the bleeding. Although a number of studies have shown that increased levels of cardiac biomarkers after SAH are associated with a worse short-term prognosis, no prospective, consecutive study has assessed the association between biomarker release and long-term outcome. We aimed to evaluate whether the cardiac biomarkers, high-sensitive troponin T (hsTnT) and N-terminal pro B-type natriuretic peptide (NTproBNP), were associated with poor 1-year neurological outcome and cerebral infarction due to delayed cerebral ischaemia (CI-DCI).

**Methods:**

In this single-centre prospective observational study, all consecutive patients admitted to our neurointensive care unit from January 2012 to December 2013 with suspected/verified SAH with an onset of symptoms <72 hours were enrolled. Blood samples for hsTnT and NTproBNP were collected during three consecutive days following admission. Patients were followed-up after 1 year using the Glasgow Outcome Scale Extended (GOSE). Poor neurological outcome was defined as GOSE ≤4.

**Results:**

One hundred and seventy seven patients with suspected SAH were admitted during the study period; 143 fulfilled inclusion criteria and 126 fulfilled follow-up. Forty-one patients had poor 1-year outcome and 18 had CI-DCI. Levels of hsTnT and NTproBNP were higher in patients with poor outcome and CI-DCI. In multivariable logistic regression modelling age, poor neurological admission status, cerebral infarction of any cause and peak hsTnT were independently associated with poor late outcome. Both peak hsTnT and peak NTproBNP were independently associated with CI-DCI.

**Conclusion:**

Increased serum levels of the myocardial damage biomarker hsTnT, when measured early after onset of SAH, are independently associated with poor 1-year outcome. Furthermore, release of both hsTnT and NTproBNP are independently associated with CI-DCI. These findings render further support to the notion that troponin release after SAH is an ominous finding. Future studies should evaluate whether there is a causal relationship between early release of biomarkers of myocardial injury after SAH and neurological sequelae.

## Background

Patients with subarachnoid haemorrhage (SAH) frequently develop cardiac complications in the acute phase after the bleeding [1]. A majority of SAH patients develop electrocardiographic changes, a substantial number have increased troponin levels and some patients develop an acute form of stress-induced cardiomyopathy [2-5], usually presenting as reversible left ventricular apical akinesia resembling Takotsubo cardiomyopathy [6]. The increased sympathetic tone with excess of circulating catecholamines seen in conjunction with the bleeding is most likely the cause of cardiac events after SAH [7-11].

A number of studies have shown that early release of the cardiac biomarkers troponin and B-type natriuretic peptide are associated with a higher risk of delayed cerebral ischaemia (DCI), cerebral infarction (CI), poor outcome and death [1, 12–15]. This has also been shown for cardiac complications, such as stress-induced cardiomyopathy [15–17]. The functional recovery after SAH is mainly studied up to 3 months after the bleeding, although many SAH patients improve their functional recovery beyond this period [18–20]. There are only a few studies reporting on the impact of cardiac biomarker release and cardiac complications on long-term outcome [21–23].

In recent years, a new method of measuring troponin has been introduced—high-sensitive troponin T (hsTnT)—with higher sensitivity and specificity for myocardial injury than previously used assays [24]. N-terminal pro B-type natriuretic peptide (NTproBNP) is a cardiac biomarker with high sensitivity and specificity for heart failure [25]. Although hsTnT and NTproBNP are increased due to other conditions in intensive care unit (ICU) patients, such as sepsis, respiratory failure and renal failure [26, 27], hsTnT and NTproBNP have a high specificity for cardiac dysfunction in SAH patients [28–30]. Therefore, the aim of this study was to evaluate whether hsTnT and/or NTproBNP are associated with poor 1-year outcome (the primary aim) and cerebral infarction due to DCI (CI-DCI; the secondary aim).

## Methods

### Study design and patient inclusion

**Fig. 1:**
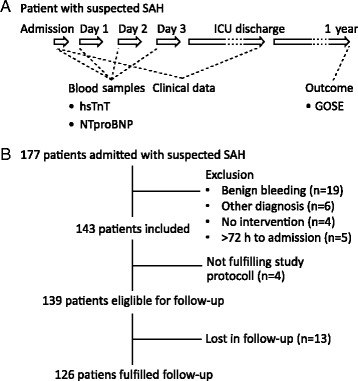
**a** Brief study protocol. Blood samples for hsTnT and NTproBNP were obtained on admission and on the following 3 days. Clinical data were recorded from admission until ICU discharge. Outcome was evaluated with the GOSE scale 1 year after admission according to a standardised procedure (see Methods for details). **b** Study flow chart. *GOSE* Glasgow Outcome Scale Extended, *hsTnT* High-sensitive troponin T, *ICU* Intensive care unit, *NTproBNP* N-terminal pro B-type natriuretic peptide, *SAH* Subarachnoid haemorrhage

This is a single-centre prospective observational study performed at the neurointensive care unit (NICU) at the Sahlgrenska University Hospital, Gothenburg. The NICU is the referral centre for 1.7 million inhabitants in the Western County Council. All referred patients are independently evaluated before admission by a consultant in neurosurgery. The study was approved by the Gothenburg Regional Ethics Committee (Ref #348-09). All patients, or patient’s next of kin, were informed about the study and were asked for consent. Study inclusion started on 1 January 2012 and ended on 31 December 2013. A brief study protocol is shown in Fig. 1a. All patients admitted with suspected SAH were eligible for inclusion. Exclusion criteria were: SAH diagnosis not confirmed, time from onset of symptoms to admission >72 hours, benign bleeding (e.g. prepontine non-aneurysmal SAH), and poor prognosis upon admission without any intervention. Time of onset of symptoms was obtained from the patients, their next of kin, or information from ambulance reports. If time of onset was unclear, it was defined as the last known time-point the patient was without symptoms. In all included patients, hsTnT and NTproBNP were measured on admission and over the following 3 days. Clinical data were recorded during ICU stay. Follow-up was performed after 1 year according to the Glasgow Outcome Scale Extended (GOSE) [31]. Follow-up was performed primarily by structured telephone interview with a standardised questionnaire [32] and secondly by a standardised letter to the patient with a questionnaire. To avoid bias in loss of follow-up, the follow-up procedure was standardised by three telephone calls to the patient. If no contact was achieved, a first letter was sent; this was followed by two telephone calls to the patient and two telephone calls to the patient’s next of kin, which was followed by a reminder letter. If no contact had been obtained after this procedure, the patient was declared as lost for follow up. Patients were treated according to the Neurocritical care consensus conference during the study period [33].

### Data collection and variable details

The primary outcome variable was GOSE ≤4 1 year after the bleeding (GOSE 1, dead; GOSE 2, vegetative state; GOSE 3, hospitalised; GOSE 4, non-independent living) [31]. The secondary outcome variable was CI-DCI verified on computed tomography (CT) or magnetic resonance imaging (MRI). This was defined as the presence of CI on CT or MRI scan of the brain within 6 weeks after SAH, or on the latest CT or MRI scan made before death within 6 weeks, not present on the CT or MRI scan between 24 and 48 hours after early aneurysm occlusion. These CT or MRI findings should not be attributable to other causes such as surgical clipping, endovascular treatment, ventricular catheter or intraparenchymal haematoma, according to the definition by Vergouwen et al. [34]. Primary predictors were peak levels of hsTnT and NTproBNP defined as the highest level during the first 72 hours after onset of symptoms. Secondary predictors were the following: a) sex, b) age, c) history of cardiovascular disease or hypertension, d) severity of SAH (graded according to World Federation of Neurological Surgeons (WFNS) grading for SAH, dichotomised to WFNS grade 1–3 and WFNS grade 4–5 [35]), e) CT grading of SAH (defined according to modified Fisher scale [36], dichotomised to modified Fisher 1–3 and modified Fisher 4), f) treatment of aneurysm (surgery or embolisation), g) haemodynamic variables and their management (blood pressure, heart rate, dose of norepinephrine during the first 3 days after admission), h) increased cerebral blood flow velocity detected by daily transcranial Doppler (TCD) examinations (defined as a peak flow velocity >200 cm/s or a diurnal increased velocity >50 cm/s in affected arteries) and i) CT verified cerebral infarction of any cause (1-year outcome analysis only).

### Biomarker analysis

hsTnT was analysed with the Roche high-sensitive troponin T assay with a coefficient of variation of 3.4 %. NTproBNP was analysed with the Elecsys assay (Roche) on Cobas platform with a coefficient of variation of 3.8 % as reported from our laboratory.

### Statistics

A *p* value <0.05 was considered significant in the analyses. Continuous variables were checked for normal distribution with inspection of histogram distribution and Shapiro–Wilks test. Student’s T-test was used to compare means of continuous normally distributed and Mann–Whitney U test was used to compare medians of non-normally distributed variables between the two groups. Fisher’s exact test was used for comparing incidences between two groups with binary variables. Spearman rank test was used to test correlation in non-normally distributed variables. Receiver operating characteristic (ROC) curves were used to determine sensitivity, specificity and cut-off levels for hsTnT and NTproBNP to predict poor outcome and CI-DCI. For comparison of levels of hsTnT and NTproBNP over time between patients with poor and good outcome, a generalised linear mixed model with gamma regression was used due to non-normal distribution of hsTnT and NTproBNP. To determine variables independently associated with late poor outcome and CI-DCI, multivariable logistic regression was used. Variables with a *p* value <0.10 on bivariate regression were selected for the multivariable analysis. All variables included in the multivariable analysis were checked for co-linearity and that data were balanced. Due to a limited number of explanatory variables allowed in the model (≈1 variable per 10 outcome observations in the poor outcome/CI-DCI group), a manual forward model building strategy was used. Variables with a previously described clinical association with the dependent variable were inserted one at a time in a reduced model. A variable was maintained in the model if the new model improved significantly, by calculating −2 log likelihood change, and if the variable itself was significant (*p* < 0.05). This was repeated until the best reduced model was obtained. The primary and remaining secondary predictors were inserted separately in the reduced model and were considered independently associated with the dependent variable if it was significant and a significant better model was obtained. Hosmer and Lemenshow test was used for goodness of fit and Nagelkerke R^2^ was used for determining explanatory degree of the model. The software used was IBM SPSS Statistics version 22.0.

## Results

A total of 710 patients were admitted to the NICU during the study period. One hundred and seventy seven patients had a suspected SAH. Thirty four patients were excluded, the main reason was a benign, prepontine bleeding (n = 19). Study protocol was not adhered to in four patients. One hundred and thirty nine patients were eligible for the 1-year follow-up, of whom 13 were lost in follow-up (Fig. 1b). Of the 126 patients fulfilling study protocol, 98 patients (78 %) were admitted on day 1, 20 patients (16 %) were admitted on day 2 and 8 patients (6 %) were admitted on day 3 after onset of symptoms.

### Patient characteristics

Patient characteristics are presented in Table 1. A total of 74 patients (59 %) were females, 38 patients (30 %) had WFNS grade 4–5 and 39 patients (31 %) had modified Fisher grade 4. In 22 patients (17 %) no aneurysm was detected. A total of 66 patients (52 %) needed acute ventricular drainage, 69 patients (55 %) were treated with embolisation and in 29 patients (23 %) an aneurysm was clipped. There were no differences regarding sex, age, surgery/embolisation, WFNS and modified Fischer grade between patients that were followed up and were lost to follow-up.

**Table 1:** Patient characteristics

	All patients	Poor outcome	CI-DCI
	(n = 126)	(n = 41)	(n = 18)
Sex			
Female	74 (59)	24 (59)	12 (67)
Male	52 (41)	17 (41)	6 (33)
Age, mean ± SD (years)	57 ± 13	61 ± 12	56 ± 14
Medical history			
Hypertension	45 (36)	16 (39)	8 (44)
Cardiovascular disease	8 (6)	4 (10)	2 (11)
Other	14 (11)	8 (20)	4 (22)
None	59 (47)	13 (32)	4 (22)
SAH severity grade			
WFNS grade 1	50 (40)	5 (12)	4 (22)
WFNS grade 2	29 (23)	8 (20)	7 (39)
WFNS grade 3	9 (7)	6 (15)	1 (6)
WFNS grade 4	20 (16)	11 (27)	5 (28)
WFNS grade 5	18 (14)	11 (27)	1 (6)
Radiological			
Modified Fischer grade 1	27 (21)	2 (5)	2 (11)
Modified Fischer grade 2	11 (9)	2 (5)	1 (6)
Modified Fischer grade 3	49 (39)	18 (44)	6 (33)
Modified Fischer grade 4	39 (31)	19 (46)	9 (50)
ICH	12 (10)	7 (17)	1 (6)
Aneurysm position			
Anterior communicating artery	33 (26)	10 (24)	3 (17)
Posterior communicating artery	14 (11)	4 (10)	5 (28)
Middle cerebral artery	21 (17)	9 (22)	1 (6)
Carotid artery	12 (10)	5 (12)	3 (17)
Other anterior circulation	8 (6)	2 (5)	1 (6)
Basilar artery	8 (6)	4 (10)	1 (6)
Other posterior circulation	7 (6)	3 (7)	2 (11)
Not found	23 (18)	4 (10)	2 (11)
Treatment			
Acute ventricular drainage	66 (52)	35 (85)	13 (72)
Embolisation	71 (56)	21 (51)	12 (67)
Surgery	30 (24)	11 (27)	3 (17)
None	25 (20)	9 (22)	3 (17)
TCD (flow velocity)			
Normal flow velocity	62 (49)	18 (44)	5 (28)
Increased flow velocity	49 (39)	18 (44)	12 (67)
Haemodynamics			
SBP on admission, mean ± SD (mmHg)	149 ± 23	147 ± 26	145 ± 25
MAP on admission, mean ± SD (mmHg)	102 ± 16	100 ± 16	99 ± 15
HR on admission, mean ± SD (beats per minute)	72 ± 15	73 ± 16	76 ± 19
SBP mean^a^, mean ± SD (mmHg)	134 ± 13	133 ± 14	133 ± 11
HR mean^a^, mean ± SD (beats per minute)	64 ± 8	68 ± 9	69 ± 7
Dose of NE mean^a^, median (IQR) (µg/kg/min)	0.01 (0-0.19)	0.10 (0-0.31)	0.09 (0-0.35)
Cerebral ischaemia			
Cerebral infarction, any cause	41 (33)	27 (66)	18 (100)
Infarction due to DCI	18 (14)	14 (34)	18 (100)
GOSE			
GOSE 1	21 (17)	21 (51)	9 (50)
GOSE 2	3 (2)	3 (7)	2 (11)
GOSE 3	7 (6)	7 (17)	1 (6)
GOSE 4	10 (8)	10 (24)	1 (6)
GOSE 5	24 (19)	0 (0)	3 (17)
GOSE 6	27 (21)	0 (0)	1 (6)
GOSE 7	11 (9)	0 (0)	1 (6)
GOSE 8	23 (18)	0 (0)	0 (0)

All values are shown as n (%) unless otherwise indicated. ^a^ Mean refers to mean value during first 3 days following admission. *CI-DCI* Cerebral infarction due to delayed cerebral ischaemia, *DCI* Delayed cerebral ischaemia, *GOSE* Glasgow Outcome Scale Extended, *HR* Heart rate, *ICH* Intracerebral haematoma, *IQR* Interquartile range, *MAP* Mean arterial pressure, *NE* norepinephrine, *SBP* Systolic blood pressure, *SD* Standard deviation, *TCD* Transcranial Doppler, *WFNS* World Federation of Neurological Surgeons

### Outcome data

Clinical variables in patients with poor outcome and CI-DCI are presented in Table 1. At 1-year follow-up, 41 patients (33 %) had poor late outcome. Fourteen patients died during ICU stay and 7 patients died after ICU discharge but before 1 year. None of the included patients died during the first 3 days after admission when the cardiac biomarkers were obtained. Patients with poor outcome were more likely to have WFNS grade 4–5 (*p* < 0.001), modified Fisher grade 4 (*p* = 0.013) or a cerebral infarction of any cause (*p* < 0.001) or caused by DCI (*p* < 0.001). They also had a higher age (*p* = 0.015), heart rate (*p* = 0.009) and dose of given norepinephrine. A total of 41 (33 %) patients had CT scan verified CI of any cause during the hospital stay. In 18 (14 %) of these patients, criteria for CI-DCI were fulfilled. Patients with CI-DCI were more likely to have increased cerebral blood flow velocities (*p* = 0.017) and had a higher heart rate (*p* = 0.028) and dose of given norepinephrine (*p* = 0.044).

### hsTNT and NTproBNP

**Fig 2.**
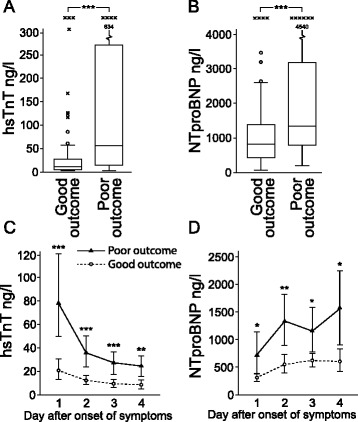
Peak levels of **a** high-sensitive troponin T (*hsTnT*) and **b** N-terminal pro B-type natriuretic peptide (*NTproBNP*) were higher in patients with poor outcome. Changes in **c** hsTnT and **d** NTproBNP over time are shown. Levels of hsTnT had its peak on day 1 after onset of symptoms followed by a daily decline both in patients with good and poor outcome. NTproBNP had its lowest levels on day 1 after onset of symptoms followed by increased levels the following days. Both hsTnT and NTproBNP levels were higher in patients with poor outcome. Day 1 refers to first 24 hours after onset of symptoms. *p < 0.05, **p < 0.01, ***p < 0.001

Peak levels of hsTnT and NTproBNP were higher in patients with poor outcome (Fig. 2a and b). hsTnT had its peak levels on admission followed by a daily decline in patients with good and poor outcome. There was a close correlation between peak hsTnT and admission hsTnT (r = 0.92). NTproBNP had lowest levels on day 1 after onset of symptoms followed by increased levels on days 2, 3 and 4 in both patients with good and poor outcome (Fig. 2c and d). Patients with CI-DCI had higher peak levels of hsTnT (*p* = 0.045; median 64 ng/l, interquartile range (IQR) 10–264 ng/l) and NTproBNP (*p* = 0.011; median 1395 ng/l, IQR 815–6770 ng/l). Peak levels of hsTnT were significantly higher in patients with WFNS grade 4–5, modified Fisher grade 4 and posterior aneurysm while peak levels of NTproBNP levels were higher in patients with WFNS grade 4–5 and posterior aneurysm. Sex, presence of intracerebral haematoma or history of cardiovascular disease did not have a significant impact on hsTnT or NTproBNP levels.

### Logistic regression analysis of variables associated with poor 1-year outcome

Bivariate logistic regression analyses of variables associated with poor outcome are presented in Table 2. Age, WFNS grade 4–5, CI of any cause, CI-DCI, modified Fisher grade 4 and intracerebral haematoma had a *p* value <0.10 and are described to have a major impact on outcome in previous studies [37] and were selected for inclusion in the reduced model. The best reduced model consisted of the variables CI of any cause, WFNS grade 4–5 and age (Table 3; Model 1); modified Fisher grade 4 and intracerebral haematoma were not significant together with the other variables. Both peak hsTnT and NTproBNP were significant in the bivariate analysis. Secondary predictors, with a *p* value <0.10, were high cerebral blood flow velocity, mean heart rate and dose of given norepinephrine. In the multivariable analysis, peak hsTnT was significant when included in the reduced model, while NTproBNP was not (Table 3; Models 2 and 3). Inclusion of mean heart rate produced a significantly better model but was not significant itself (*p* = 0.056). None of the other secondary predictors were significant in the multivariable model.

**Table 2:** Bivariate logistic regression; 1-year poor outcome (GOSE ≤4)

Category	Variable	OR	95 % CI	*p* value
Background data	Age, per year	1.04	1.01–1.07	0.018
	Sex	1.01	0.47–2.16	0.976
	History of hypertension	1.23	0.57–2.67	0.590
Admission data	WFNS grade 4–5	4.99	2.20–11.34	<0.001
	Modified Fischer grade 4	2.81	1.27–6.20	0.011
	Intracerebral haematoma	3.29	0.98–11.11	0.055
	Posterior aneurysm	1.72	0.57–5.20	0.336
Treatment	Surgery	1.34	0.54–3.33	0.530
TCD	Increased flow velocities	1.41	0.64–3.10	0.398
Cerebral infarction	Cerebral infarction, any cause	8.09	3.47–18.84	<0.001
Cardiovascular data	SBP on admission	0.99	0.98–1.01	0.474
	MAP on admission	0.99	0.96–1.01	0.341
	Heart rate on admission	1.01	0.98–1.03	0.652
	SBP mean^a^	0.99	0.96–1.02	0.685
	Heart rate mean^a^	1.06	1.01–1.11	0.011
	Given NE mean^a^, per 0.10 μg/kg/h	1.13	1.01–1.27	0.040
Biomarker data	hsTnT peak, per 100 ng/l	1.88	1.28–2.78	0.001
	NTproBNP peak, per 1000 ng/l	1.22	1.05–1.42	0.009

^a^Mean refers to mean value during first 3 days following admission. *CI* Confidence interval, *GOSE* Glasgow Outcome Scale Extended, *hsTnT* High-sensitive troponin T, *MAP* Mean arterial pressure, *NE* norepinephrine, *NTproBNP* N-terminal pro B-type natriuretic peptide, *OR* Odds ratio, *SBP* Systolic blood pressure, *TCD* Transcranial Doppler, *WFNS* World Federation of Neurological Surgeons

**Table 3:** Multivariable regression models; 1-year poor outcome (GOSE ≤4)

	Variable	OR	95 % CI	*p* value	−2LL	Sig –2LL change	Nagelkerke R^2^
Model 1	Cerebral infarction, any cause	11.43	4.15–31.5	<0.001	110.28		0.447
	WFNS grade 4–5	6.57	2.39–18.04	<0.001			
	Age, per year	1.05	1.01–1.10	0.009			
Model 2	Cerebral infarction, any cause	11.40	3.99–32.57	<0.001	99.05	<0.001	0.528
	WFNS grade 4–5	3.58	1.21–10.67	0.022			
	Age, per year	1.06	1.01–1.10	0.013			
	hsTnT peak, per 100 ng/l	1.59	1.10–2.29	0.013			
Model 3	Cerebral infarction, any cause	9.74	3.48–27.25	<0.001	106.63	0.056	0.462
	WFNS 4–5	5.72	2.06–15.87	0.001			
	Age, per year	1.05	1.01–1.09	0.021			
	NTproBNP peak, per 1000 ng/l	1.10	0.97–1.24	0.140			

*−2LL* −2 log likelihood, *CI* Confidence interval, *GOSE* Glasgow Outcome Scale Extended, *hsTnT* High-sensitive troponin T, *NTproBNP* N-terminal pro B-type natriuretic peptide, *OR* Odds ratio, *WFNS* World Federation of Neurological Surgeons

### Logistic regression analysis of variables associated with CI-DCI

Bivariate logistic regression analyses of variables associated with CI-DCI are presented in Table 4. TCD detected increased flow velocity and modified Fisher had a *p* value <0.10 and are described to have an association with CI-DCI and were therefore selected for inclusion in the reduced model. The best reduced model included TCD detected increased flow velocities only (Table 5; Model 1). Peak hsTnT and NTproBNP as well as mean heart rate and dose of given norepinephrine had a *p* value <0.10 and were included in the multivariable analysis. Both peak hsTnT and NTproBNP were significant when included together with TCD detected increased flow velocities (Table 5; Models 2 and 3).

**Table 4:** Bivariate logistic regression; CI-DCI

Category	Variable	OR	95 % CI	*p* value
Background data	Age, per year	0.75	0.95–1.03	0.750
	Sex	0.67	0.24–1.93	0.647
	History of hypertension	2.00	0.73–5.47	0.177
Admission data	WFNS grade 4–5	1.19	0.41–3.44	0.751
	Modified Fischer grade 4	2.60	0.94–7.18	0.065
	Posterior aneurysm	1.46	0.36–5.90	0.594
Treatment	Surgery	0.53	0.13–2.04	0.354
TCD	Increased flow velocities	3.89	1.27–11.94	0.017
Cardiovascular data	SBP on admission	0.99	0.97–1.01	0.380
	MAP on admission	0.98	0.95–1.01	0.289
	Heart rate on admission	1.02	0.99–1.05	0.191
	SBP mean^a^	0.99	0.95–1.03	0.658
	Heart rate mean^a^	1.07	1.00–1.13	0.033
	Given NE mean^a^, per 0.10 μg/kg/h	1.14	1.00–1.30	0.048
Biomarker data	hsTnT peak, per 100 ng/l	1.23	1.01–1.50	0.040
	NTproBNP peak, per 1000 ng/l	1.12	1.02–1.23	0.018

^a^Mean refers to mean value during first 3 days following admission. *CI* Confidence interval, *CI-DCI* Cerebral infarction due to delayed cerebral ischaemia, *hsTnT* High-sensitive troponin T, *MAP* Mean arterial pressure, *NE* norepinephrine, *NTproBNP* N-terminal pro B-type natriuretic peptide, *OR* Odds ratio, *SBP* Systolic blood pressure, *TCD* Transcranial Doppler, *WFNS* World Federation of Neurological Surgeons

**Table 5:** Multivariable regression models; CI-DCI

		OR	95 % CI	*p* value	−2LL	Sig –2LL change	Nagelkerke R^2^
Model 1	TCD, increased flow velocities	3.84	1.33–11.05	0.013	96.70		0.092
Model 2	TCD, increased flow velocities	3.86	1.31–11.34	0.015	92.73	0.046	0.144
	hsTnT peak, per 100ng/l	1.24	1.01–1.53	0.043			
Model 3	TCD, increased flow velocities	3.40	1.15–10.04	0.027	91.79	0.026	0.153
	NTproBNP peak, per 1000ng/l	1.11	1.00–1.23	0.044			

*−2LL* −2 log likelihood, *CI* Confidence interval, *CI-DCI* Cerebral infarction due to delayed cerebral ischaemia, *hsTnT* High-sensitive troponin T, *NTproBNP* N-terminal pro B-type natriuretic peptide, *OR* Odds ratio, *TCD* Transcranial Doppler, *WFNS* World Federation of Neurological Surgeons

### Sensitivity and specificity analysis

The best cut-off value of peak hsTnT to predict poor late outcome was 51 ng/l which had a sensitivity of 56 % and a specificity of 84 %, while the best cut-off value of peak NTproBNP was 1230 ng/l which had a sensitivity of 58 % and a specificity of 71 %. Area under the curve was 0.74 for hsTnT (*p* < 0.001) and 0.70 for NTproBNP (*p* < 0.001). Using the cut-off value 51 ng/l, hsTNT had an odds ratio of 5.4 in a bivariate logistic regression of poor late outcome. To predict CI-DCI, the best cut-off value of peak hsTnT was 69 ng/l which had a sensitivity of 50 % and a specificity of 82 %. The best cut-off value of peak NTproBNP was 1250 ng/l with a sensitivity of 66 % and a specificity of 68 %. Area under the curve was 0.64 (*p* = 0.047) for hsTnT and 0.69 for NTproBNP (*p* = 0.011).

## Discussion

In the present prospective study, we searched for risk factors associated with poor 1-year neurological outcome and the development of CI-DCI in patients with SAH. In this aspect, we were particularly interested in evaluating the role of the cardiac biomarkers indicative of myocardial injury or heart failure (hsTnT and NTproBNP) in the early phase after admission. The major finding was that increased levels of hsTnT, early upon admission to NICU, were independently associated with poor late outcome when adjusted for other risk factors known to affect long-term outcome. In addition, we could demonstrate that both cardiac biomarkers hsTnT and NTproBNP were independently associated with CI-DCI.

Patients with poor neurological long-term outcome were more likely to have higher levels of both hsTnT and NTproBNP. Furthermore, in the bivariate logistic regression, peak hsTnT and NTproBNP were both strongly associated with poor long-term outcome. In addition, hsTnT was independently associated with poor 1-year outcome when adjusting for important predictors for poor outcome such as cerebral infarction, poor neurological status on admission (WFNS grade 4–5) and age. Using the cut-off value suggested from the ROC analysis, patients with a peak hsTnT >51 ng/l had a fivefold increased risk for poor late neurological outcome, i.e. hsTnT had the same impact in the analysis as poor neurological status on admission. Although the predictive value of hsTnT to predict poor outcome was only fair to moderate, the specificity was high (84 %). Thus, poor outcome after SAH is multifactorial, and a low hsTnT does not exempt the patient from a poor prognosis but a high hsTnT indicates a high risk of poor outcome. One could argue that our finding of an association between increased cardiac biomarkers and poor long-term outcome is an epiphenomenon. However, as increased troponins were independently associated with poor outcome when adjusted for neurological status upon admission and cerebral infarction we suggest that a causative relationship is possible.

The findings of increased cardiac biomarker levels in patients with poor neurological long-term outcome were accompanied by higher heart rates and requirement for higher doses of catecholamines during the first 3 days after admission. We have previously shown that increased levels of hsTnT and NTproBNP are strongly associated with stress-induced cardiomyopathy after SAH, which is also described by other groups [28–30]. Thus, the systemic haemodynamics were, most likely, more compromised in patients with poor neurological long-term outcome. One could speculate whether such a haemodynamic instability also impaired cerebral perfusion in these compromised patients, particularly since it has been suggested that autoregulation of cerebral blood flow might be impaired in SAH [38]. This is supported by a recent study showing that patients with increased troponins had decreased focal and global cerebral perfusion [39]. Both troponin release and a higher heart rate after SAH are associated with a higher sympathetic tone [40, 41]. Thus, the higher sympathetic activity, with excessive release of endogenous norepinephrine, and the higher doses of exogenously administered norepinephrine to the patients with poor neurological long-term outcome might have induced a higher cerebral metabolic oxygen demand compared to patients with good neurological long-term outcome. Indeed, it has been shown that norepinephrine increases cerebral metabolism, when the blood–brain barrier is injured, which is seen after SAH [42,43]. Cardiac dysfunction with haemodynamic instability in combination with an increased cerebral metabolic demand and impaired cerebral autoregulation could contribute to a cerebral oxygen supply/demand mismatch with an increased risk of cerebral infarction. This hypothesis is supported by our finding that peak hsTnT and NTproBNP were both independently associated with CT-detected CI-DCI. However, this finding must be interpreted with caution, as only 18 patients had CI-DCI. Interestingly, biomarker-detected myocardial damage was also significantly associated with poor long-term outcome even when adjusting for CT-verified cerebral infarctions. One could speculate that this could be explained by diffuse cerebral damage, not detected by ordinary CT scan, due to cerebral oxygen supply/demand mismatch, causing poor late neurologic outcome. Future studies should evaluate whether there is a causal relationship between early release of biomarkers of myocardial injury after SAH and late neurological outcome.

The novel finding of this study is that increased troponins are independently associated with long-term poor outcome when adjusting for known variables of poor outcome. Previous studies have shown that increased levels of troponin-I are associated with death and poor prognosis at 3 months [1, 12]. BNP and NTproBNP are associated with a worse short-term outcome and cerebral infarction after SAH [13-15, 44]. Cardiac complications such as left ventricular regional wall motion abnormalities were associated with cerebral infarction and dependent living at 3 months after SAH in a large recent multicentre study [15], which is also supported by previous smaller studies [17, 22, 44, 45]. Furthermore, an increased heart rate has been described as being associated with poor 3-month outcome [46], a finding that was also demonstrated in the present study. Although most neurological complications are seen within 3 months after onset of symptoms, there are several studies reporting that patients improve beyond this point [18–20]. Factors associated with poor long-term outcome are poor neurological status on admission, cerebral infarction and higher age [47, 48]. These factors are known ominous clinical findings and were also found to be significantly associated with poor long-term outcome in the present study. However, no prospective consecutive study has previously reported on the association between release of cardiac biomarkers or cardiac complications and long-term outcome. The few studies available are either non-consecutive, retrospective or have small sample sizes, and show contradictory results [21–23]. The finding of the present study—that cardiac troponin release is independently associated with poor long-term outcome—gives further support to the notion that troponin release after SAH is an ominous finding.

The main limitation with this study is the limited number of patients and the fact that this was a single-centre study. The relatively low number of patients with poor outcome ruled out the possibility of performing a multivariable analysis in which all variables of interest were included. However, instead of inappropriately including too many variables, we used a clinically relevant model building. Performing echocardiography in all patients would have been valuable, as this could have added one more dimension to the data. The strengths of this study are the consecutive and prospective design and the number of patients included in the follow-up (91 %). We also used the latest assay of troponin measurement, hsTnT, which has a higher sensitivity and specificity for detecting cardiac damage than previously used assays. In addition, high-sensitive assays are recommended by current guidelines [49] and hsTnT will probably be the dominating biomarker for early detection of myocardial injury.

## Conclusion

In conclusion, we found that increased serum levels of the myocardial injury biomarker hsTnT, when taken early after admission, is independently associated with poor 1-year neurological outcome in patients with SAH. In addition, we could demonstrate that increased serum levels of the cardiac biomarkers hsTnT and NTproBNP are independently associated with CI-DCI. These findings further highlight the importance of increased troponins after SAH. Future studies should evaluate whether there is a causal relationship between early release of biomarkers of myocardial injury after SAH and late neurological outcome.

## Key messages

- Increased serum levels of the myocardial injury biomarker hsTnT, upon admission, is independently associated with poor long-term outcome in SAH, when adjusted for known predictors of poor long-term outcome.
- Increased serum levels of hsTnT and the myocardial function biomarker NTproBNP, upon admission, are both independently associated with cerebral infarction due to delayed cerebral ischaemia.
- These findings give further support to the notion that an early release of the cardiac biomarkers hsTnT and NTproBNP are ominous findings associated with neurological sequelae after SAH.

## Abbreviations

CI: Cerebral infarction
CI-DCI: Cerebral infarction due to delayed cerebral ischaemia
CT: Computer tomography
DCI: Delayed cerebral ischaemia
GOSE: Glasgow Outcome Scale Extended
hsTnT: High-sensitive troponin T
ICU: Intensive care unit
IQR: Interquartile range
MRI: Magnetic resonance imaging
NICU: Neurointensive care unit
NTproBNP: N-terminal pro B-type natriuretic peptide
ROC: Receiver operating characteristic
SAH: Subarachnoid haemorrhage
TCD: Transcranial Doppler
WFNS: World Federation of Neurological Surgeons
